# Computational Prediction of RNA-Binding Proteins and Binding Sites

**DOI:** 10.3390/ijms161125952

**Published:** 2015-11-03

**Authors:** Jingna Si, Jing Cui, Jin Cheng, Rongling Wu

**Affiliations:** Center for Computational Biology, National Engineering Laboratory for Tree Breeding, College of Biological Sciences and Technology, Beijing Forestry University, Beijing 100083, China; cuijing19900925@163.com (J.C.); chengjin@bjfu.edu.cn (J.C.); rwu@bjfu.edu.cn (R.W.)

**Keywords:** RNA-binding proteins (RBPs), RNA-binding site, bioinformatics, prediction, macromolecular docking

## Abstract

Proteins and RNA interaction have vital roles in many cellular processes such as protein synthesis, sequence encoding, RNA transfer, and gene regulation at the transcriptional and post-transcriptional levels. Approximately 6%–8% of all proteins are RNA-binding proteins (RBPs). Distinguishing these RBPs or their binding residues is a major aim of structural biology. Previously, a number of experimental methods were developed for the determination of protein–RNA interactions. However, these experimental methods are expensive, time-consuming, and labor-intensive. Alternatively, researchers have developed many computational approaches to predict RBPs and protein–RNA binding sites, by combining various machine learning methods and abundant sequence and/or structural features. There are three kinds of computational approaches, which are prediction from protein sequence, prediction from protein structure, and protein-RNA docking. In this paper, we review all existing studies of predictions of RNA-binding sites and RBPs and complexes, including data sets used in different approaches, sequence and structural features used in several predictors, prediction method classifications, performance comparisons, evaluation methods, and future directions.

## 1. Introduction

Approximately 6%–8% of proteins are RNA-binding proteins (RBPs). These RBPs play an important part in gene expression and regulation. Due to study limitations, only a few types of RBPs have been identified such as HuR, AUF1, TTP, TIA1, and CUGBP2. These RBPs perform essential roles in various biological processes such as mRNA stability [1], stress responses [2], cell cycle, tumor differentiation [3], apoptosis, and gene regulation at the transcriptional and post-transcriptional levels [4]. Determining the three-dimensional (3D) structures of protein–RNA complexes facilitates the identification of physiochemical properties and biological interactions.

Experimental methods (e.g., nuclear magnetic resonance spectroscopy (NMR) [5] and X-ray crystallography [6]) typically used for protein–RNA complex structure determination are expensive, time-consuming and labor-intensive. To date, 2274 protein–RNA complex structures determined by experimental methods have been deposited in the Protein Data Bank (PDB) database [7]. The number of protein–RNA complexes in the PDB database is significantly fewer than that which exists in nature. Given the large numbers of nucleic acid and protein sequences that exist, improved knowledge of how protein–RNA interactions occur could help us to recognize functional information.

**Figure 1:**
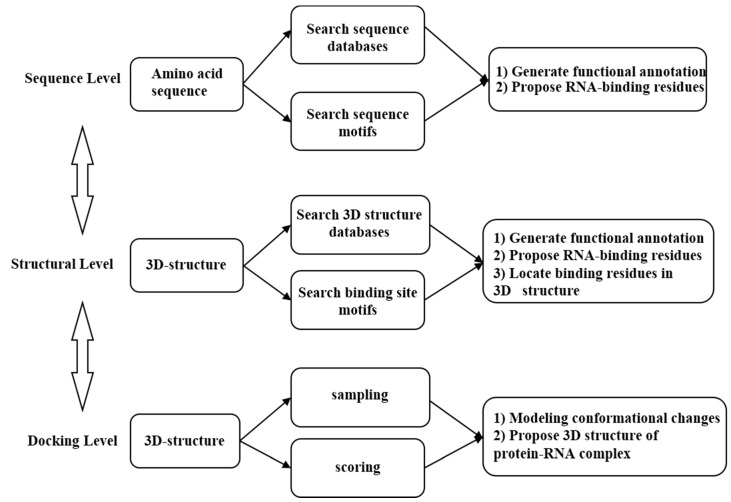
Strategies for RNA-binding site and RBP prediction.

To achieve this goal, it is necessary to develop computational approaches which can reliably and rapidly identify RAN-binding proteins or sites. In contrast with experimental methods, computational tools could inexpensively and quickly identify RNA-binding sites and RBPs, which would be useful and helpful in studying protein–RNA interactions [8]; however, those methods based only on amino acid sequence information are difficult since organisms are highly complex. Several methods have been developed which focus on predicting RNA-binding sites and determining whether a protein–RNA complex exists. The majority of previous studies have focused on prediction approaches for RNA-binding sites and RBPs based on sequence similarity [9,10,11,12]. The query protein sequences were searched against databases; if the homologous sequences were known RNA-binding proteins, the query protein was regarded as an RNA-binding protein. Similarly, RNA-binding residues and sites in the query sequence could be detected. For another, methods based on predicted structural and sequence information are the most often used computational approaches to identify RNA-binding sites or RBPs. If the 3D structure of a target protein is known, the prediction based on structure information was carried out to distinguish RBPs [13,14,15]. It is believed that the structural similarity could provide more reliable and in-depth prediction consequence. Another technique is docking, a method started from the components coordinates, and aimed at modelling interaction conformation of macromolecular complexes [16]. Many protein–protein docking tools have been reported, but no specific RNA–protein docking method exists [17]. Several protein–protein docking programs accept RNA and protein coordinates as inputs to generate RBPs, such as HADDOCK [18], GRAMM [19], HEX [20], PatchDock [21], and FTDock [22]. The above strategies for RNA-binding site and RBP prediction are summarized in Figure 1.

Although the methodology for predicting protein–protein interactions and protein–DNA interactions are well established [23,24], analyses of computational approaches used to identify protein–RNA interactions are lacking [8,17]. In this review, we discuss computational approaches for predicting RBPs and RNA-binding sites based on protein sequences or known protein 3D structures. Moreover, RNA–protein complex docking methods were discussed. We summarize detailed information of these computational tools, including various vectors based on sequence and/or structure, datasets used in the algorithm, performance comparison, machine learning methods, and so on. In particular, we summarize those available web servers for RNA-binding sites and RBP prediction, which are convenient for scientists. Finally, the future directions and several implications have been discussed, which can aid in method development.

## 2. Development of Computational Methods for Prediction of RNA-Binding Site

### 2.1. Data Set

The sequence and structure of protein–RNA complexes are available from PDB database and other specific protein–RNA interaction databases (Available online: http://pridb.gdcb.iastate.edu/) [25]. We analyzed several previous studies and summarize the datasets and methods used, which are listed in detail in Table 1. Of all existing datasets, RB344 is the largest and contains 344 non-redundant RBPs with at least 30% sequence identity [26]. In several studies, authors employed the same dataset to compare the advantages and disadvantages of various methods. In particular, Cheng *et al.* [27] constructed a novel PRIPU dataset which differed from previous datasets. The PRIPU dataset contained positive and unlabeled, but not negative samples. Such negative samples sometimes are not necessarily genuine negative samples and may even be unknown positive samples.

**Table 1:** Commonly used data sets for RNA-binding sites identification.

ID	Reference	Publication Year	Notes
PRIPU dataset	[27]	2015	The dataset contains positive and unlabeled examples, which is an innovation because previous ones usually have negative samples. Such negative samples are not real negative samples, some even may be unknown positive samples
^a^ RB344	[26]	2015	344 RNA binding proteins, almost entirely non-redundant at 30% sequence identity
RB172	[28]	2014	172 protein entries with sequence identity of less than 25%
RB75	[8]	2012	75 RNP complexes released between 1 January and 28 April 2011 from PDB database ^b^, non-redundant at 40% sequence identity
RB199	[25,29]	2011	Extracted dataset (May 2010) from PDB database. Proteins with >30% sequence identity or structures with resolution worse than 3.5 Å were removed
RB164	[30]	2010	The data were downloaded from RsiteDB. After removing protein and RNA chains with sequence identity above 25% and 60%, respectively, 205 non-redundant protein–RNA chains in 164 complexes were obtained
RB86	[31]	2008	86 RNA-binding protein chains were collected for training and fivefold cross validation
RB147	[32]	2007	Adding novel RNA-binding complexes since 2006, based on RB109
RB109	[33]	2006	109 RNA–protein complexes extracted from structures of known RNA–protein complexes solved by X-ray crystallography in the PDB. Proteins with >30% sequence identity or structures with resolution worse than 3.5 Å were removed

^a^ RB: Abbreviation of RNA-binding dataset; ^b^ PDB: Protein Data Bank.

RNA-binding residues are determined using two definitions: (i) a residue with any atom within 3–6 Å of any atom in a nucleotide; and (ii) residues involved in hydrophobic, electrostatic interactions with nucleotides, van der Waals, or hydrogen-bonding [25]. Residues satisfying these definitions are considered to be RNA-binding residues. As with protein–DNA complexes and protein–protein complexes, similar sequences in protein–RNA interactions are eliminated before dataset construction. Generally, sequences with similarities greater than 30%–40% are considered redundant. Clustering programs such as blastclust (available from NCBI), CD-HIT [34], and the PISCES web server are used to generate a non-redundant dataset.

### 2.2. Feature Selection for RNA-Binding Residues and Protein Predictors

Many features have been used to identify RBPs and binding sites. There are three kinds of features here, which are structure-based features, sequence-based features, chemical and physical features. The commonly used features summarized here include amino acid composition, sequence similarity, evolutionary information, accessible surface area (ASA), predicted secondary structures (SSs), hydrophobicity, electrostatic patches, cleft sizes, and other global protein features. Details of these features are shown as follows.

#### 2.2.1. Sequence-Based Features

##### Amino Acid Composition

One of the most commonly used features of protein sequence is protein amino acid composition, not only in protein–protein interaction site prediction, but also in RNA-binding site prediction. The 20 amino acids exhibit various properties based on the presence of hydrophobic residues (G, F, L, M, A, I, P, V), polar residues (Q, T, S, N, C, Y, W), and charged residues (H, R, K, E, D) [35]. One of the encoding methods are based on the physicochemical properties of the various residue types. The hydrophobic, polar, charged and residues are encoded as (1 0), (0 1), and (0 0), respectively. Particularly, the positively-charged RNA backbone is usually more likely to combine with the negatively-charged residues, as shown in previous studies [36]. The other encoding method is standard binary encoding, which encodes each amino acid as a 20-dimensional binary vector, such as E (0 0 0 1 0 0 0 0 0 0 0 0 0 0 0 0 0 0 0 0), F (0 0 0 0 1 0 0 0 0 0 0 0 0 0 0 0 0 0 0 0), A (1 0 0 0 0 0 0 0 0 0 0 0 0 0 0 0 0 0 0 0 0),…, and Y (0 0 0 0 0 0 0 0 0 0 0 0 0 0 0 0 0 0 0 1).

##### Sequence Similarity

Sequence similarity (also referred to as sequence conservation) is frequently used for RNA-binding site prediction. The BLAST and PSI-BLAST programs are used to compare the similarities among various protein sequences. Generally, multiple sequence alignment (MSA) were obtained by comparing query sequences against the NCBI non-redundant database and were used to calculate each residue’s sequence similarity score. A number of conservation scoring tools are available including relative entropy, von Neumann entropy, Shannon entropy, and Scorecons.

##### Evolutionary Information

Evolutionary information has often been introduced in functional site predictors in recent studies, including RNA-binding site prediction. Previous studies showed that position-specific scoring matrix (PSSM) (an important form of evolutionary information) greatly improved the performance of RBPs prediction. PSSMs were used widely in pervious prediction studies because they provide the likelihood of a particular residue substitution based on evolutionary information.

#### 2.2.2. Structure-Based Features

##### The Secondary Structure (SS)

The secondary structure (SS) provides local and geometric patterns, which can be obtained in two ways: One is that the protein structure is available and real SS could be calculated using SS assignment approach such as DSSPcont [37,38], the other is that the protein structure is unavailable and predicted SS could be obtained using SS predicted algorithm such as PSIPRED [38,39,40]. SS has been employed as an encoding feature in several studies to predict RNA-binding residues [41,42].

##### Accessible Surface Area (ASA)

RNA-binding residues tend to be exposed and interact with proteins, so calculation of solvent accessibility would be helpful in RNA-binding sites prediction. The relative ASA could be calculated using NACCESS [43,44], while the protein structure is available. It is worth pointing out that the relative ASA could not be calculated when the DNA molecule was absent. Residues with ASA value greater than 5% are defined as surface accessible residues.

#### 2.2.3. Chemical and Physical Features

##### Hydrophobicity

Hydrophobicity, which represents the proportion of residues repelled by water, is frequently used by RNA-binding site predictors. Hydrophobicity scale was defined with numerical value for each amino acid [45].

##### Electrostatic Patches

A protein surface status can be described by electrostatic patches. Generally, nucleic acid-binding interfaces are more likely to be positively charged electrostatic patches [46]. Electrostatic patches can be computed using GRASP [47], GRASS [48], or the web server PFplus (PatchFinderPlus; Available online: http://pfp.technion.ac.il) [49].

##### Cleft Size

Cleft size is an important feature because the largest cleft on a protein surface tends to be where the protein active site is located [50]. The charge, dipole, and quadrupole moments can also be used to adequately recognize RBPs [51].

### 2.3. Prediction Methods

The computational methods used in previous studies to identify RBPs or RNA-binding sites can be divided into three aspects: (1) the use of sequence-based prediction methods when structure is unknown and sequence is known; (2) prediction methods based on structure when the query protein structure has been resolved; and (3) modeling using a docking method when the query structure is unknown. These three approaches are detailed below.

#### 2.3.1. Sequences-Based Methods

##### Sequence-Based Methods for RNA-Binding Site Prediction

The number of known protein–RNA complex structures is few, so prediction methods which use only sequence information play an important role. Previously, Jeong *et al.* [52] introduced a predictor for RNA-binding sites using predicted secondary structure and amino acid type, and employingan artificial neural network. Subsequently, Terribilini *et al.* [33] contributed RNABindR, which is a classical method to train naive Bayes (NB) classifiers to predict RNA-binding sites. The RB109 dataset is listed in Table 1. Wang and Brown developed the BindN tool, which is a predictor of RNA- and DNA-binding sites [9]. The sequence features used in this method include molecular mass, hydrophobicity index, and the side chain pKa value. In addition, the evolutionary information was added to predictors, especially in the form of PSSMs. Pprint was developed by Kumar *et al.* [31], which combined evolutionary information (PSSM) and support vector machines (SVMs) and improved RNA-binding site and residue predictions significantly. Wang *et al.* [53] used SVM and PSSM profiles coupled with predicted SS and PSI-BLAST profiles in the PRINTR method to obtain improved performance. Tong *et al.* [54] introduced RISP, which is a hybrid RNA-binding site predictor which uses SVMs in conjunction with PSSMs and achieved a 61.0% increase in sensitivity and an 83.3% increase in specificity. A similar method, RNAProB, using SVM and a novel smoothed PSSM encoding method, was developed by Cheng *et al.* [55] and it gave better performance than the then current state-of-the-art systems. In 2010, Li *et al.* [56] constructed a novel method, employing evolutionary PSSM and structure-derived features to predict RNA-binding residues, which led to significant improvement. Liu *et al.* [30] proposed a novel classification system that combined sequence/structure-based features and interaction propensity, which is a novel interacting feature. In addition, a novel machine learning method (random forest) was used. Furthermore, Liu *et al.* compared their method with previous methods (e.g., RNAProB, PPRint, BindN and RNABindR) and achieved enhanced performance. Zhang *et al.* [57] presented an RNA-binding residue predictor using solvent accessibility, predicted SS, evolutionary conservation and sequence information. RNABindRPlus [58] is a recently developed predictor which obviously improved prediction reliability, which combines sequence homology and machine learning methods. Recently, Cheng *et al.* [27] developed a predictor (PRIPU) for protein–RNA interactions; the most important difference between this and original methods is that only positive and unlabeled samples are used in PRIPU, not negative samples.

##### Sequence-Based Methods for RNA-Binding Proteins (RBPs) Prediction

Han *et al.* [36] explored the SVM machine learning method to predict RBPs directly based on their primary sequence. The dataset in this work contained 447 RBPs and 4881 non-RBPs. The prediction accuracy was 40.0% and 99.9% for snRBPs and non-snRBPs, respectively, indicating the need for a sufficient number of proteins to train the SVMs. Shao *et al.* [59] developed a predictor to predict RNA-binding proteins with SVM methods using sequence characteristics. Similar to RNA-binding site prediction, evolutionary information was introduced to improve the performance of RBP predictions. Kumar *et al.* [60] exploited RNApred which combined binding residues and PSSM profiles and the SVM method to discriminate RBPs and non-RBPs. Another voting system was used to identify RBPs [42]. Zhao *et al.* developed SPOT for prediction of RBPs using a fold recognition method, which is freely available on the internet for academic users (Table 2).

#### 2.3.2. Structure-Based Methods

##### Structure-Based Methods for RNA-Binding Site Prediction

When the structure of the query protein is available and employed in the prediction system, the prediction became more reliable. There are a number of structure-based RNA-binding site prediction methods. Kim *et al.* [13] developed KYG method, which uses sequence profiles, doublets of spatially close residues, a number of binding scores, and combinations. Chen and Lim [61] developed a predictor based on structure information including electrostatics, evolution, and geometry. The disparate cleft and the surface patch were considered to be RNA-binding site. Subsequently, PRIP [62] was created, which exploited structural and topological information (retention coefficient, betweenness-centrality, accessible surface area and PSI-BLAST profile) and used two machine learning methods (SVM and naive Bayes classifiers). Towfic *et al.* [63] contributed Struct-NB, which used structural features to predict RNA-binding sites by combining a naive Bayes classifier. Recently, two predictors based on structure were proposed. RBRDetector [64], which uses evolutionary and microenvironmental features as inputs, combines feature- and template-based strategies to improve predictions of RNA-binding residues. The other predictor compares each template patch with surrounding patches and uses the accumulated distances as structural features [26].

##### Structure-Based Methods for RBP Prediction

Zhao *et al.* [15] introduced a predictor for RNA-binding domains based on structure information, which combined RNA binding affinity and relative structural similarity. SPOT-Seq-RNA [65] is a template-based structure prediction package which integrates RBP, RNA-binding residue, and protein–RNA complex structure prediction. RBPs and protein–RNA complexes are often modeled using the docking method.

#### 2.3.3. Protein–RNA Complex Docking

Research on protein 3D structure modeling has become increasingly complex. Modeling structures of a protein–RNA complex is very important to help us understand the mechanisms of interaction. Several docking techniques used to predict protein–RNA complexes rely on known RNA and protein structures. There are no protein–RNA interaction docking algorithms, most reported docking techniques are modified from those protein-ligand interaction and protein–protein interaction docking softwares by employing certain energy/scoring function that fitted for protein–RNA interactions. For example, Katchalski-Katzir *et al.* [19] developed a low-resolution docking program, which requires specific scoring functions for different ligands. In the modeling progress, the program performs a six-dimensional search through the rotation of a ligand molecule and the rigid body translation and generates decoys. Gabb *et al.* [22] employed the FTDOCK program, which not only accepts protein–protein docking, but also accepts nucleic acid molecules. Ritchie and Kemp [20] introduced Hex, which enables protein–nucleic acid and protein–protein docking. The decoy scoring method contains electrostatics and shape-matching but does not have a special function for protein–RNA complexes. The method of Haddock [18] enables various molecules (e.g., nucleic acids, proteins and other small molecules) for docking, which using biochemical and biophysical characteristics as inputs. Recently, Tuszynska and Bujnicki [66] developed QUASI-RNP and DARS-RNP, which use statistical and quasi-chemical reference states to score protein–RNA decoys.

### 2.4. Prediction Algorithms

Almost all popular machine learning methods have been used for prediction of RNA-binding sites or RBPs. Generally, the machine learning methods obtain satisfactory performance with valid sequence- and/or structure-based features participation. The machine learning methods frequently used for RNA-binding research include SVMs [27,67], artificial neural networks (ANN) [68], Bayesian networks [29,67], and random forest [12,69]. Puton *et al.* [8] have attempted a meta-predictor of RNA-binding residues based on three of the highest ranked sequence-based primary predictors. This meta-predictor outperforms most other predictors. The template-based approach is another algorithm to predict structure of protein–RNA complex when a template structure is available. This method recognizes the putative RBPs by structurally aligning the query protein to RBPs with known structures. SPARKS X [15] is a program which predicts structure based on template-based structure. Similarly, TIM-align [70] is a structural alignment program.

### 2.5. Evaluation and Performance of Various Predictors

#### 2.5.1. Performance Measures

The parameters commonly used to assess RNA-binding sites and RBP prediction performance include sensitivity, accuracy, strength, specificity, F-measure, precision, the Matthews correlation coefficient (MCC), and area under the receiver operating characteristic curve (AUC), these parameters are detailed listed in Table 3.

For the formula presented in Table 3, TP represents true positives which are correctly predicted RNA-binding residues; FP indicates false positives which are mistakenly predicted RNA-binding residues; TN denotes true negatives which are correctly predicted non-RNA-binding residues; and FN means false negatives which are wrongly predicted non-RNA-binding residues. Due to the imbalance between positive sample and negative sample, the MCC is regarded as proper measurement to evaluate the overall performance. “MCC = 0” means completely random prediction, and “MCC = 1” indicates perfect prediction. Higher value of MCC (between 0 and 1) represents better prediction accuracy. Another widely used measurement is the receiver operating characteristic (ROC) curve, especially in the comparison of several predictors. The *x*-axis of ROC curve represents the true positive rate and the *y*-axis denotes the false positive rate. The larger the area under the curve (AUC), the better the method.

#### 2.5.2. Comparison of Various Prediction Methods

The prediction results of existing methods for RNA-binding sites and RBP predictions are summarized in Table 4. The accuracy of most predictions is approximately 60%–80% and the specificity and sensitivity of these methods range widely. Each method has its own specialty because of the various datasets, input features, and algorithms. Three main datasets are listed in Table 4 including RB75, RB172, and RB344. Several original studies [8,28,71] compared several predictors independently based on the unified dataset and their results are summarized in this manuscript. The MCC is always considered an unbiased measurement and has been calculated in most methods, which helps significantly when comparing the performance among these methods. Subsequently, a meta-predictor that combines three predictors has been developed and has satisfactory performance [8].

#### 2.5.3. Collection of Web Servers of RBPs and RNA-Binding Site Predictors

Many researchers provide web servers when they develop novel methods to predict RNA-binding sites and RBPs. Several protein–RNA complex docking programs are also available. We collected the URLs which are divided into sequence- and structure-based predictors and docking methods (Table 2). We have tested every web server and labeled them with “○” or “X” if the web server is available or not, respectively, and noted if the approach is aimed at predicting binding sites or RBPs. Actually, web servers could provide easy-to-use tools to the community. Users could understand the algorithm and conveniently obtain prediction results using web servers. Meanwhile, developers could continually modify their methods with users’ feedback.

## 3. Conclusions and Future Perspectives

Due to the significant biological roles of several RNA types, RNA-binding site prediction has become more and more important in the area of protein functional site prediction. Prediction accuracy has improved significantly during the past decades and a number of web servers are available to experimental scientists. Nevertheless, the current predictors require further research to improve their effectiveness due to shortcomings.

Three outstanding issues face efforts to predict RNA-binding sites and RBPs. The first important issue is how to distinguish DNA-binding sites from RNA-binding sites. Generally, the prediction approaches that use templates are more effective than those using machine learning methods for distinguishing RBPs from DNA-binding proteins. Conversely, for those RBPs that could not detect successfully using template-based methods, several machine learning methods can detect RNA-binding residues. Therefore, combining the strengths of two approaches has the potential to obtain better performance of RNA-binding site and RBP prediction. The second important issue is that which vectors contribute more and which ones offer less to the mature predictor in machine learning methods remains unclear. It is certain that selection of novel and effective features could be one of the most important concepts in RBPs and RNA-binding site prediction. The third issue is that all existing protein–RNA docking approaches do not take into account conformational changes that may occur in the combination process of protein and RNA molecules. The ability to model the 3D RNA structure using several RNA folding simulations [72,73,74] and accommodating those methods to refold RNA fragments to simulate protein–RNA interaction and optimize minimum energy would be useful [75,76,77,78,79]. Rother *et al.* [80] successfully combined RNA and protein 3D structures into a unified modeling method. Moreover, further comparison studies are required to adequately evaluate the advantages and disadvantages of various methods.

**Table 2:** A general selection of Web servers of RNA-binding sites and protein prediction and protein–RNA complex docking.

Methods	URLs	References	Available	Seq/Struc/Docking	Sites/Protein
PRIPU	http://admis.fudan.edu.cn/projects/pripu.htm	Cheng *et al.* (2015) [27]	○	seq	site
RNABindRPlus	http://einstein.cs.iastate.edu/RNABindRPlus/	Walia *et al.* (2014) [58]	○		site
CatRAPID omics	http://s.tartaglialab.com/catrapid/omics	Agostini *et al.* (2013) [81]	○		site
SRCPred	http://tardis.nibio.go.jp/netasa/srcpred	Fernandez *et al.* (2011) [29]	○		site
SPOT	http://sparks.informatics.iupui.edu	Zhao *et al.* (2011) [15]	X		protein
PRBR	http://www.cbi.seu.edu.cn/PRBR/	Ma *et al.* (2011) [12]	○		site
RNAPred	http://www.imtech.res.in/raghava/rnapred/	Kumar *et al.* (2011) [60]	○		protein
RPISeq	http://pridb.gdcb.iastate.edu/RPISeq/	Muppirala *et al.* (2011) [82]	○		site
BindN+	http://bioinfo.ggc.org/bindn+/	Wang *et al.* (2010) [11]	○		site
NAPS	http://prediction.bioengr.uic.edu/	Carson *et al.* (2010) [81]	X		site
PiRaNhA	http://bioinformatics.sussex.ac.uk/PIRANHA/	Murakami *et al.* (2010) [10]	○		site
PRNA	http://www.sysbio.ac.cn/datatools.asp	Liu *et al.* (2010) [56]	X		site
RNA	http://mcgill.3322.org/RNA/	Li *et al.* (2010) [55]	X		site
RISP	http://grc.seu.edu.cn/RISP	Tong *et al.* (2008) [54]	X		site
PRINTR	http://210.42.106.80/printr/	Wang *et al.* (2008) [53]	X		site
PPRInt	http://www.imtech.res.in/raghava/pprint/	Kumar *et al.* (2008) [52]	○		site
RNABindR	http://bindr2.gdcb.iastate.edu/RNABindR/	Terribilini *et al.* (2007) [32]	○		site
BindN	http://bioinfo.ggc.org/bindn/	Wang and Brown (2006) [9]	○		site
SVMProt	http://jing.cz3.nus.edu.sg/cgi-bin/svmprot.cgi	Han *et al.* (2004) [36]	X		protein
RBPDetector	http://ibi.hzau.edu.cn/rbrdetector	Yang *et al.* (2014) [64]	○	struc	site
SPOT-Seq-RNA	http://sparks-lab.org/server/SPOT-Seq-RNA/	Yang *et al.* (2014) [65]	X		protein
DRNA	http://sparks.informatics.iupui.edu/yueyang/DFIRE/dRdR-DB-service	Zhao *et al.* (2011) [15]	X		protein
OPRA	Program available upon request from the authors	Perez-Cano and Fernandez-Recio (2010) [14]	○		site
PRIP	http://www.qfab.org/PRIP	Maetschke *et al.* (2009) [62]	X		site
KYG	http://cib.cf.ocha.ac.jp/KYG/	Kim *et al.* (2006) [13]	X		protein
DARS-RNP and QUASI-RNP	http://www.genesilico.pl/RNP/	Tuszynska and Bujnicki (2011) [66]	○	docking	complex
PatchDock	http://bioinfo3d.cs.tau.ac.il/PatchDock/index.html	Schneidman-Duhovny *et al.* (2005) [21]	○		complex
Haddock	http://www.nmr.chem.uu.nl/haddock/; http://haddock.science.uu.nl/services/HADDOCK	Dominguez *et al.* (2003) [18]	○		complex
Hex	http://hex.loria.fr/; http://hexserver.loria.fr/	Ritchie and Kemp (2000) [20]	○		complex
FTDock (3D-Dock)	http://www.sbg.bio.ic.ac.uk/docking/	Gabb *et al.* (1997) [22]	○		complex
GRAMM	http://vakser.bioinformatics.ku.edu/main/resources_gramm1.03.php	Katchalski-Katzir *et al.* (1992) [19]	○		complex

○: denotes the URL is available now; X: means the URL is not available nowadays; URLs: Abbreviations of UniformResourceLocators.

**Table 3:** Evaluation parameters.

Parameter	Meaning	Expression
Accuracy (ACC)	Percentage of correct prediction	$Accuracy=\frac{TP+TN}{TP+TN+FP+FN}$ ^a^
Sensitivity	Percentage of correctly predicted positive	$Sensitivity=\frac{TP}{TP+FN}$
Specificity	Percentage of correctly predicted negative	$Specifcity=\frac{TN}{TN+FP}$
Strength	Mean value of the sum of sensitivity and specificity	$Strength=\frac{Sensitivity+Specifcity}{2}$
MCC	Matthews correlation coefficient	$\text{MCC}=\frac{\left(TP\times TN\right)-(FN\times FP)}{\sqrt{\left(TP+FN)\times (TN+FP)\times (TP+FP)\times (TN+FN\right)}}$
Precision	Positive predictive rate	$Precision=\frac{TP}{TP+FP}$
F-measure	The harmonic mean of sensitivity and specificity	$\text{F}-\text{measure}=\;\frac{2\;\times \;Presion\;\times \;Sensitivity}{\text{Presion}+\text{Sensitivity}}$
AUC ^b^	Probability that a classifier will rank a randomly chosen positive instance higher than a randomly chosen negative one	$AUC=\frac{\sum_{i-1}^{n}T_{i}}{nT}$

^a^ TP = True positive number; TN = True negative number; FP = False positive number; FN = False negative number; ^b^ In AUC formulation, *i* takes on values from 1 to *n*, *T* is the total number of positives in the test set, and *T_i_* is the number of positives that score higher than the *i*th highest scoring negative.

**Table 4:** Performance of the state-of-the-art methods for RNA-binding site prediction.

Methods	Data Set	Performance								Reference	Feature
		ACC	SEN	SPE	AUC	MCC	Strength	F-Measure	Precision		
PiRaNhA	RB75	-	-	-	0.822	0.435	-	-	-	[8]	Sequence-based
PPRInt	RB75	-	-	-	0.779	0.339	-	-	-	[8]	
	RB172	0.71			-	0.25	0.66	-	-	[28]	
	RB344	0.70	0.45	0.82	0.68	0.28	-	0.49	0.53	[26]	
BindN	RB75	-	-	-	0.733	0.297	-	-	-	[8]	
	RB172	0.75	-	-	-	0.23	0.64	-	-	[28]	
BindN+	RB75	-	-	-	0.821	0.397	-	-	-	[8]	
	RB172	0.79	-	-	-	0.34	0.71	-	-	[28]	
	RB344	0.72	0.32	0.89	0.68	0.26	-	0.41	0.56	[26]	
RNABindR	RB75	-	-	-	0.708	0.317	-	-	-	[8]	
RNABindR v2.0	RB172	0.66	-	-	-	0.27	0.69	-	-	[28]	
PRBR	RB75	-	-	-	N/A ^a^	0.294	-	-	-	[8]	
NAPS	RB75	-	-	-	0.679	0.215	-	-	-	[8]	
	RB172	0.66	-	-	-	0.17	0.61	-	-	[28]	
RNAProB	RB172	0.82	-	-	-	0.22	0.60	-	-	[28]	
KYG *	RB75	-	-	-	N/A	0.382	-	-	-	[8]	Structure-based
DRNA *	RB75	-	-	-	N/A	0.382	-	-	-	[8]	
	RB344	0.75	0.21	0.94	N/A	0.22	-	0.31	0.54	[26]	
OPRA *	RB75	-	-	-	N/A	0.296	-	-	-	[8]	
Ren’s method	RB344	0.68	0.48	0.76	0.68	0.26	-	0.48	0.48	[26,83]	
Meta-predictor ^b^	RB75	-	-	-	0.835	0.460	-	-	-	[8,34]	

^a^ N/A—not available; MCC—Matthews Correlation Coefficient; AUC—area under curve; SEN—sensitivity; SPE—specificity; ^b^ Meta-predictor developed based on top three sequence-based methods according to authors benchmark (PiRaNhA, PPRInt and BindN+); * The meta-predictor is composed of those methods labeled with asterisk.

## References

[1] Jacobs Anderson J.S., Parker R. (2000). Computational identification of cis-acting elements affecting post-transcriptional control of gene expression in Saccharomyces cerevisiae. Nucleic Acids Res..

[2] Abdelmohsen K., Kuwano Y., Kim H.H., Gorospe M. (2008). Posttranscriptional gene regulation by RNA-binding proteins during oxidative stress: Implications for cellular senescence. Biol. Chem..

[3] Saunus J.M., French J.D., Edwards S.L., Beveridge D.J., Hatchell E.C., Wagner S.A., Stein S.R., Davidson A., Simpson K.J., Francis G.D. (2008). Posttranscriptional regulation of the breast cancer susceptibility gene BRCA1 by the RNA binding protein HuR. Cancer Res..

[4] Noller H.F. (2005). RNA structure: Reading the ribosome. Science.

[5] Orengo C.A., Michie A.D., Jones S., Jones D.T., Swindells M.B., Thornton J.M. (1997). CATH—A hierarchic classification of protein domain structures. Structure.

[6] Ponting C.P., Schultz J., Milpetz F., Bork P. (1999). SMART: Identification and annotation of domains from signalling and extracellular protein sequences. Nucleic Acids Res..

[7] Berman H.M., Westbrook J., Feng Z., Gilliland G., Bhat T.N., Weissig H., Shindyalov I.N., Bourne P.E. (2000). The Protein Data Bank. Nucleic Acids Res..

[8] Puton T., Kozlowski L., Tuszynska I., Rother K., Bujnicki J.M. (2012). Computational methods for prediction of protein–RNA interactions. J. Struct. Biol..

[9] Wang L., Brown S.J. (2006). BindN: A web-based tool for efficient prediction of DNA and RNA binding sites in amino acid sequences. Nucleic Acids Res..

[10] Murakami Y., Spriggs R.V., Nakamura H., Jones S. (2010). PiRaNhA: A server for the computational prediction of RNA-binding residues in protein sequences. Nucleic Acids Res..

[11] Wang L., Huang C., Yang M.Q., Yang J.Y. (2010). BindN+ for accurate prediction of DNA and RNA-binding residues from protein sequence features. BMC Syst. Biol..

[12] Ma X., Guo J., Wu J., Liu H., Yu J., Xie J., Sun X. (2011). Prediction of RNA-binding residues in proteins from primary sequence using an enriched random forest model with a novel hybrid feature. Proteins.

[13] Kim O.T., Yura K., Go N. (2006). Amino acid residue doublet propensity in the protein-RNA interface and its application to RNA interface prediction. Nucleic Acids Res..

[14] Perez-Cano L., Solernou A., Pons C., Fernández-Recio J. (2010). Structural prediction of protein-RNA interaction by computational docking with propensity-based statistical potentials. Pac. Symp. Biocomput..

[15] Zhao H., Yang Y., Zhou Y. (2011). Structure-based prediction of RNA-binding domains and RNA-binding sites and application to structural genomics targets. Nucleic Acids Res..

[16] Moreira I.S., Fernandes P.A., Ramos M.J. (2010). Protein–protein docking dealing with the unknown. J. Comput. Chem..

[17] Tuszynska I., Matelska D., Magnus M., Chojnowski G., Kasprzak J.M., Kozlowski L.P., Dunin-Horkawicz S., Bujnicki J.M. (2014). Computational modeling of protein–RNA complex structures. Methods.

[18] Dominguez C., Boelens R., Bonvin A.M. (2003). HADDOCK: A protein–protein docking approach based on biochemical or biophysical information. J. Am. Chem. Soc..

[19] Katchalski-Katzir E., Shariv I., Eisenstein M., Friesem A.A., Aflalo C., Vakser I.A. (1992). Molecular surface recognition: Determination of geometric fit between proteins and their ligands by correlation techniques. Proc. Natl. Acad. Sci. USA.

[20] Ritchie D.W., Kemp G.J. (2000). Protein docking using spherical polar Fourier correlations. Proteins.

[21] Schneidman-Duhovny D., Inbar Y., Nussinov R., Wolfson H.J. (2005). PatchDock and SymmDock: Servers for rigid and symmetric docking. Nucleic Acids Res..

[22] Gabb H.A., Jackson R.M., Sternberg M.J. (1997). Modelling protein docking using shape complementarity, electrostatics and biochemical information. J. Mol. Biol..

[23] Si J., Zhao R., Wu R. (2015). An overview of the prediction of protein DNA-binding sites. Int. J. Mol. Sci..

[24] Wichadakul D., McDermott J., Samudrala R. (2009). Prediction and integration of regulatory and protein-protein interactions. Methods Mol. Biol..

[25] Lewis B.A., Walia R.R., Terribilini M., Ferguson J., Zheng C., Honavar V., Dobbs D.S. (2011). PRIDB: A Protein–RNA interface database. Nucleic Acids Res..

[26] Ren H., Shen Y. (2015). RNA-binding residues prediction using structural features. BMC Bioinform..

[27] Cheng Z., Zhou S., Guan J. (2015). Computationally predicting protein-RNA interactions using only positive and unlabeled examples. J. Bioinf. Comput. Biol..

[28] Nagarajan R., Gromiha M.M. (2014). Prediction of RNA binding residues: An extensive analysis based on structure and function to select the best predictor. PLoS ONE.

[29] Fernandez M., Kumagai Y., Standley D.M., Sarai A., Mizuguchi K., Ahmad S. (2011). Prediction of dinucleotide-specific RNA-binding sites in proteins. BMC Bioinform..

[30] Cheng C.-W., Su E.C.-Y., Hwang J.-K., Sung T.-Y., Hsu W.-L. (2008). Predicting RNA-binding sites of proteins using support vector machines and evolutionary information. BMC Bioinform..

[31] Ahmad S., Sarai A. (2011). Analysis of electric moments of RNA-binding proteins: Implications for mechanism and prediction. BMC Struct. Biol..

[32] Terribilini M., Sander J.D., Lee J.H., Zaback P., Jernigan R.L., Honavar V., Dobbs D. (2007). RNABindR: A server for analyzing and predicting RNA-binding sites in proteins. Nucleic Acids Res..

[33] Petrey D., Honig B. (2003). GRASP2: Visualization, surface properties, and electrostatics of macromolecular structures and sequences. Methods Enzymol..

[34] Si J., Zhang Z., Lin B., Schroeder M., Huang B. (2011). MetaDBSite: A meta approach to improve protein DNA-binding sites prediction. BMC Syst. Biol..

[35] Bartlett G.J., Porter C.T., Borkakoti N., Thornton J.M. (2002). Analysis of catalytic residues in enzyme active sites. J. Mol. Biol..

[36] Han L.Y., Cai C.Z., Lo S.L., Chung M.C., Chen Y.Z. (2004). Prediction of RNA-binding proteins from primary sequence by a support vector machine approach. RNA.

[37] Kabsch W., Sander C. (1983). Dictionary of protein secondary structure: Pattern recognition of hydrogen-bonded and geometrical features. Biopolymers.

[38] Carter P., Andersen C.A., Rost B. (2003). DSSPcont: Continuous secondary structure assignments for proteins. Nucleic Acids Res..

[39] Si J.N., Yan R.X., Wang C., Zhang Z., Su X.D. (2009). TIM-Finder: A new method for identifying TIM-barrel proteins. BMC Struct. Biol..

[40] Karypis G. (2006). YASSPP: Better kernels and coding schemes lead to improvements in protein secondary structure prediction. Proteins.

[41] Jones D.T. (1999). Protein secondary structure prediction based on position-specific scoring matrices. J. Mol. Biol..

[42] Peng C.R., Liu L., Niu B., Lv Y.L., Li M.J., Yuan Y.L., Zhu Y.B., Lu W.C., Cai Y.D. (2011). Prediction of RNA-binding proteins by voting systems. J. Biomed. Biotechnol..

[43] Yu X., Cao J., Cai Y., Shi T., Li Y. (2006). Predicting rRNA-, RNA-, and DNA-binding proteins from primary structure with support vector machines. J. Theor. Biol..

[44] Hubbard S.J., Thornton J.M. (1993). NACCESS Computer Program.

[45] Kyte J., Doolittle R.F. (1982). A simple method for displaying the hydropathic character of a protein. J. Mol. Biol..

[46] Stawiski E.W., Gregoret L.M., Mandel-Gutfreund Y. (2003). Annotating nucleic acid-binding function based on protein structure. J. Mol. Biol..

[47] Nayal M., Hitz B.C., Honig B. (1999). GRASS: A server for the graphical representation and analysis of structures. Protein Sci..

[48] Shazman S., Celniker G., Haber O., Glaser F., Mandel-Gutfreund Y. (2007). Patch Finder Plus (PFplus): A web server for extracting and displaying positive electrostatic patches on protein surfaces. Nucleic Acids Res..

[49] Laskowski R.A., Luscombe N.M., Swindells M.B., Thornton J.M. (1996). Protein clefts in molecular recognition and function. Protein Sci..

[50] Jeong E., Chung I.F., Miyano S. (2004). A neural network method for identification of RNA-interacting residues in protein. Genome Inform..

[51] Terribilini M., Lee J.H., Yan C., Jernigan R.L., Honavar V., Dobbs D. (2006). Prediction of RNA binding sites in proteins from amino acid sequence. RNA.

[52] Kumar M., Gromiha M.M., Raghava G.P. (2008). Prediction of RNA binding sites in a protein using SVM and PSSM profile. Proteins.

[53] Wang Y., Xue Z., Shen G., Xu J. (2008). PRINTR: Prediction of RNA binding sites in proteins using SVM and profiles. Amino Acids.

[54] Tong J., Jiang P., Lu Z.H. (2008). RISP: A web-based server for prediction of RNA-binding sites in proteins. Comput. Methods Programs Biomed..

[55] Li Q., Cao Z., Liu H. (2010). Improve the prediction of RNA-binding residues using structural neighbours. Protein Pept. Lett..

[56] Liu Z.P., Wu L.Y., Wang Y., Zhang X.S., Chen L. (2010). Prediction of protein–RNA binding sites by a random forest method with combined features. Bioinformatics.

[57] Zhang T., Zhang H., Chen K., Ruan J., Shen S., Kurgan L. (2010). Analysis and prediction of RNA-binding residues using sequence, evolutionary conservation, and predicted secondary structure and solvent accessibility. Curr. Protein Pept. Sci..

[58] Walia R.R., Xue L.C., Wilkins K., El-Manzalawy Y., Dobbs D., Honavar V. (2014). RNABindRPlus: A predictor that combines machine learning and sequence homology-based methods to improve the reliability of predicted RNA-binding residues in proteins. PLoS ONE.

[59] Shao X., Tian Y., Wu L., Wang Y., Jing L., Deng N. (2009). Predicting DNA- and RNA-binding proteins from sequences with kernel methods. J. Theor. Biol..

[60] Kumar M., Gromiha M.M., Raghava G.P. (2011). SVM based prediction of RNA-binding proteins using binding residues and evolutionary information. J. Mol. Recognit..

[61] Chen Y.C., Lim C. (2008). Predicting RNA-binding sites from the protein structure based on electrostatics, evolution and geometry. Nucleic Acids Res..

[62] Maetschke S.R., Yuan Z. (2009). Exploiting structural and topological information to improve prediction of RNA–protein binding sites. BMC Bioinform..

[63] Towfic F., Caragea C., Gemperline D.C., Dobbs D., Honavar V. (2010). Struct-NB: Predicting protein–RNA binding sites using structural features. Int. J. Data Min. Bioinform..

[64] Yang X.X., Deng Z.L., Liu R. (2014). RBRDetector: Improved prediction of binding residues on RNA-binding protein structures using complementary feature- and template-based strategies. Proteins.

[65] Yang Y., Zhao H., Wang J., Zhou Y. (2014). SPOT-Seq-RNA: Predicting protein-RNA complex structure and RNA-binding function by fold recognition and binding affinity prediction. Methods Mol. Biol..

[66] Tuszynska I., Bujnicki J.M. (2011). DARS-RNP and QUASI-RNP: New statistical potentials for protein-RNA docking. BMC Bioinform..

[67] Choi S., Han K. (2011). Prediction of RNA-binding amino acids from protein and RNA sequences. BMC Bioinform..

[68] Walia R.R., Caragea C., Lewis B.A., Towfic F., Terribilini M., El-Manzalawy Y., Dobbs D., Honavar V. (2012). Protein-RNA interface residue prediction using machine learning: An assessment of the state of the art. BMC Bioinform..

[69] Pan X., Zhu L., Fan Y.X., Yan J. (2014). Predicting protein–RNA interaction amino acids using random forest based on submodularity subset selection. Comput. Biol. Chem..

[70] Zhang Y., Skolnick J. (2005). TM-align: A protein structure alignment algorithm based on the TM-score. Nucleic Acids Res..

[71] Zhao H., Yang Y., Zhou Y. (2013). Prediction of RNA binding proteins comes of age from low resolution to high resolution. Mol. Biosyst..

[72] Denesyuk N.A., Thirumalai D. (2013). Coarse-grained model for predicting RNA folding thermodynamics. J. Phys. Chem. B.

[73] Ding F., Sharma S., Chalasani P., Demidov V.V., Broude N.E., Dokholyan N.V. (2008). Ab initio RNA folding by discrete molecular dynamics: From structure prediction to folding mechanisms. RNA.

[74] Das R., Baker D. (2007). Automated *de novo* prediction of native-like RNA tertiary structures. Proc. Natl. Acad. Sci. USA.

[75] Chan D.S., Lee H.M., Yang F., Che C.M., Wong C.C., Abagyan R., Leung C.H., Ma D.L. (2010). Structure-based discovery of natural-product-like TNF-α inhibitors. Angew. Chem. Int. Ed. Engl..

[76] Leung C.H., Zhong H.J., Yang H., Cheng Z., Chan D.S., Ma V.P., Abagyan R., Wong C.Y., Ma D.L. (2012). A metal-based inhibitor of tumor necrosis factor-α. Angew. Chem. Int. Ed. Engl..

[77] Ma D.L., Lin S., Leung K.H., Zhong H.J., Liu L.J., Chan D.S., Bourdoncle A., Mergny J.L., Wang H.M., Leung C.H. (2014). An oligonucleotide-based label-free luminescent switch-on probe for RNA detection utilizing a G-quadruplex-selective iridium(III) complex. Nanoscale.

[78] Ma D.L., Liu L.J., Leung K.H., Chen Y.T., Zhong H.J., Chan D.S., Wang H.M., Leung C.H. (2014). Antagonizing STAT3 dimerization with a rhodium(III) complex. Angew. Chem. Int. Ed. Engl..

[79] Zhong H.J., Lu L., Leung K.H., Wong C.C.L., Peng C., Yan S.C., Leung C.H. (2015). An iridium(III)-based irreversible protein–protein interaction inhibitor of BRD4 as a potent anticancer agent. Chem. Sci..

[80] Rother K., Rother M., Boniecki M., Puton T., Bujnicki J.M. (2011). RNA and protein 3D structure modeling: Similarities and differences. J. Mol. Model..

[81] Agostini F., Zanzoni A., Klus P., Marchese D., Cirillo D., Tartaglia G.G. (2013). catRAPID omics: A web server for large-scale prediction of protein-RNA interactions. Bioinformatics.

[82] Muppirala U.K., Honavar V.G., Dobbs D. (2011). Predicting RNA-protein interactions using only sequence information. BMC Bioinform..

[83] Carson M.B., Langlois R., Lu H. (2010). NAPS: A residue-level nucleic acid-binding prediction server. Nucleic Acids Res..

